# Spatial distribution and associated factors of public healthcare infrastructure in tourism-oriented environments: evidence from Southwest China in the context of residential tourism

**DOI:** 10.3389/fpubh.2026.1912761

**Published:** 2026-08-25

**Authors:** Hong Zhao, Wei Wei, Shenghua Yu, Chaoju Wang

**Affiliations:** 1 Department of Tourism Management, South China University of Technology, Guangzhou, China; 2 Guizhou Minzu University, Guiyang, China

**Keywords:** health geography, healthcare accessibility, public healthcare infrastructure, residential tourism, spatial distribution

## Abstract

**Introduction:**

Public healthcare infrastructure is essential for ensuring equitable access to medical services, particularly in mountainous regions characterized by uneven development and tourism-oriented spatial environments. This study examines the spatial distribution of public healthcare infrastructure in Southwest China from a health geography perspective with particular attention to accessibility, spatial inequality and residential tourism contexts.

**Methods:**

A cross-sectional spatial analytical framework was applied to examine public healthcare infrastructure in Southwest China. Spatial analysis methods and the geographic detector model were employed to identify distribution patterns and associated factors. Residential tourism was operationalized through tourism-related spatial proxy variables rather than direct measures of tourist mobility or seasonal residence.

**Results:**

Public healthcare infrastructure exhibited a significant clustered distribution with high-density areas concentrated in Chengdu, Chongqing, Guiyang and Kunming and extensive low-density areas located in remote mountainous regions. Road network density showed the strongest explanatory power, followed by A-level scenic spot density and residential density. Interaction effects generally exceeded the explanatory power of individual variables with the interaction between road network density and A-level scenic spot density being the strongest. Traditional Village density showed negligible independent explanatory power.

**Conclusion:**

The distribution of public healthcare infrastructure in Southwest China reflects pronounced core-periphery inequality in facility concentration and the combined spatial associations of transportation connectivity, residential concentration, regional development and tourism-oriented environments. The findings extend the contextual application of health geography to residential tourism settings and provide a spatial screening basis for differentiated healthcare infrastructure planning. They should be interpreted as cross-sectional spatial associations rather than evidence of population mobility or seasonal healthcare demand.

## 1 Introduction

Public healthcare infrastructure constitutes a fundamental component of regional health systems, playing a critical role in ensuring equitable access to medical services and improving population health outcomes (1, 2). Its spatial distribution affects healthcare accessibility, service efficiency and social equity (3, 4). In recent decades, rapid urbanization, regional development and changing population distribution have increased healthcare demand (5, 6) and raised concerns regarding the spatial organization and allocation of healthcare infrastructure (7, 8).

From the perspective of health geography, healthcare provision is spatially embedded and geographically uneven. Geographic location, transportation conditions, population distribution, environmental constraints and socio-economic development jointly shape access to healthcare services and may generate regional disparities in service utilization and health outcomes (9–11). In this study, health geography provides a framework centered on three interrelated dimensions: the spatial concentration of healthcare facilities, geographic accessibility shaped by transportation and residential distribution, and place-based associations between healthcare infrastructure and regional environmental, socio-economic and tourism-related contexts. This framework is particularly relevant to regions characterized by complex terrain and uneven development.

Southwest China, comprising Yunnan, Guizhou, Sichuan and Chongqing, provides a representative context in which natural constraints, socio-economic disparities and tourism-oriented development converge (12, 13). Its mountainous and plateau landscapes create barriers to transportation and public service provision, while uneven economic development contributes to marked urban–rural and core-periphery differences in healthcare infrastructure (14, 15). At the same time, the region contains abundant ethnic cultural heritage (16) and tourism resources (17) and has become an important area for tourism-related development. These characteristics make Southwest China suitable for examining how accessibility, regional inequality and tourism-related spatial environments are associated with the distribution of public healthcare infrastructure.

Residential tourism refers to tourism-related residence involving longer stays, repeated visits or semi-permanent residence in destination areas (18), rather than conventional short-term sightseeing or travel undertaken primarily for medical treatment. Tourists, retirees and seasonal residents in such settings may temporarily use local public services including healthcare services (19). Residential tourism therefore provides a relevant context for examining healthcare infrastructure in tourism-oriented regions (20). However, this study does not directly measure residential tourist population size, length of stay, seasonal intensity or individual mobility. Instead, it adopts a cross-sectional design to examine whether tourism-related spatial environments are associated with the distribution of public healthcare infrastructure.

Despite the importance of public healthcare infrastructure in mountainous and tourism-oriented regions, previous research has primarily explained facility distribution through permanent population density (21), socio-economic development (22) and transportation conditions (23). Although these factors remain essential, less attention has been paid to whether tourism-oriented spatial environments are associated with healthcare facility distribution and how they interact with residential and regional development conditions, particularly in areas where residential spaces, tourism destinations and public service system overlap. This issue is especially relevant in Southwest China, where complex terrain, uneven development and tourism-oriented growth coexist. A clearer understanding of these spatial associations is necessary to determine whether conventional healthcare geography patterns remain dominant and whether tourism-related environments provide additional explanatory relevance. Building on this research need, the present study operationalizes tourism-related environments through four measurable indicators: A-level scenic spot density, park and square density, homestay accommodation density and traditional village density. These indicators do not measure residential tourism populations but represent complementary spatial conditions that may support longer stays, repeated visits and temporary residence. A-level scenic spots represent destination attractions that may encourage repeated or extended visits; parks and squares provide everyday leisure and social spaces; homestay accommodation offers the most direct spatial indication of tourism-oriented temporary lodging and residence; and traditional villages represent culturally distinctive rural living environments that may support place-based residential tourism experiences. These variables are therefore interpreted collectively as environmental proxies for residential tourism contexts rather than as direct or equivalent measures of long-stay tourist populations.

Accordingly, this study addresses four research questions: (1) What are the spatial distribution characteristics of public healthcare infrastructure in the residential tourism context of Southwest China? (2) Which environmental, socio-economic and tourism-related factors are most strongly with its spatial distribution? (3) How do transportation connectivity, residential distribution, socio-economic conditions and tourism-related spatial indicators interact in explaining healthcare infrastructure distribution? (4) How can these spatial patterns and associations be interpreted through the health geography perspectives of accessibility, spatial inequality and place-based interaction?

This study therefore aims to examine the spatial distribution and associated factors of public healthcare infrastructure in Southwest China using spatial analysis and geographic detector methods. Three working hypotheses are proposed. First, public healthcare infrastructure is expected to exhibit a clustered rather than random spatial pattern, with higher concentrations in urbanized and accessible areas. Second, transportation connectivity, residential density and tourism-related spatial indicators are expected to exhibit stronger explanatory associations than natural environmental factors. Third, interactions between transportation connectivity and tourism-related spatial indicators are expected to enhance the explanatory power of individual factors. The study contributes cross-sectional spatial evidence on healthcare infrastructure distribution and extends the application of health geography to residential tourism environments by integrating accessibility, spatial inequality and place-based tourism-related contexts. It does not propose a new causal theory or directly measure population mobility.

## 2 Literature review

Public healthcare infrastructure, including general hospitals, primary healthcare institutions, community health service centers and township health centers, forms an essential component of public health service systems (24, 25). Its scale and spatial distribution influence healthcare accessibility (26), service efficiency (27), population health outcomes and health equity (28). Although healthcare infrastructure expansion has become an important policy priority (29), substantial spatial inequalities remain between urban and rural areas (30) and between economically developed and less developed regions (31). Existing research can be broadly grouped into three related perspectives: healthcare infrastructure as a foundation for equitable public service provision (32); spatial accessibility shaped by facility location (33), population distribution (34) and transportation conditions (35); resource allocation efficiency in relation to regional development capacity (36, 37). These perspectives have generated important insights, but they generally explain healthcare demand through relatively stable resident populations and conventional socio-economic conditions.

The development of GIS and spatial analysis techniques has shifted healthcare infrastructure research from simple location mapping toward the analysis of spatial accessibility (38), regional inequality (39) and place-based service provision (40). From a health geography perspective, healthcare facilities are understood as components of a spatially embedded public service system whose distribution is associated with population distribution (41), transportation networks (42), environmental constraints (43) and regional development conditions (44). Previous studies have identified significant clustering and regional disparities in healthcare infrastructure linked to population distribution, economic development and infrastructure conditions.

Methodologically, this field has evolved from descriptive spatial analysis to accessibility assessment and explanatory spatial modeling. Nearest neighbor analysis (45), spatial autocorrelation (46) and kernel density estimation (47) are commonly used to identify clustering, dispersion and concentration patterns. Network-based (48) and catchment-area (49) approaches further assess the relationship between facility location and population service coverage. More recently, spatial regression (50) and geographic detector (51) methods have been employed to explain spatial heterogeneity and interaction effects among determinants. Empirical findings generally show that healthcare facilities concentrate in economically developed and accessible urban areas (52), while remote and mountainous regions often experience lower facility concentration because of transport constraints and limited development capacity (53). This evolution highlights the value of combining spatial pattern identification with factor explanation.

Beyond conventional determinants, population mobility (54) and tourism-related residence (55) have increasingly been discussed as potential influences on public service demand. Residential tourism differs from short-term tourism, medical tourism and wellness tourism because it involves longer stays, repeated visits or semi-permanent residence by tourists, retirees and seasonal residents (56, 57). These populations may temporarily use local healthcare, transportation and community services, making residential tourism a relevant context for examining public healthcare infrastructure.

Existing residential tourism research has mainly focused on lifestyle migration (58), well-being (18), housing (59), destination development (60) and local socio-economic change (61). Its implications for the spatial allocation of public healthcare infrastructure remain less developed. The relationship can be understood through potential service demand and spatial co-location: tourism-related spaces such as scenic spots, homestay accommodation areas, parks and cultural landscapes may overlap with residential areas, transportation networks and public service facilities. However, the present study does not directly observe residential tourist numbers, length of stay or seasonal mobility. Tourism-related indicators are therefore treated as spatial proxies for tourism-oriented environments rather than direct measures of mobile populations.

Previous research related to this topic remains fragmented across several fields. Healthcare infrastructure studies have mainly explained spatial disparities through resident population, economic development, transportation connectivity and urban–rural inequality. Tourism and health research has more often focused on medical tourism, wellness tourism, tourist health risks or healthcare accessibility for visitors. Meanwhile, residential tourism studies have examined long-stay visitors, retirees, seasonal residents, and destination development, but have rarely connected these issues with the spatial distribution of public healthcare infrastructure. Existing research provides a useful foundation, although the integration of healthcare infrastructure, tourism-related spatial environments and residential tourism contexts within unified spatial explanatory framework remains relatively limited. Building on previous research, the present study combines spatial pattern analysis with geographic detector methods to examine how environmental, socio-economic, residential and tourism-related indicators are associated with public healthcare infrastructure distribution in Southwest China.

## 3 Study area

**Figure 1:**
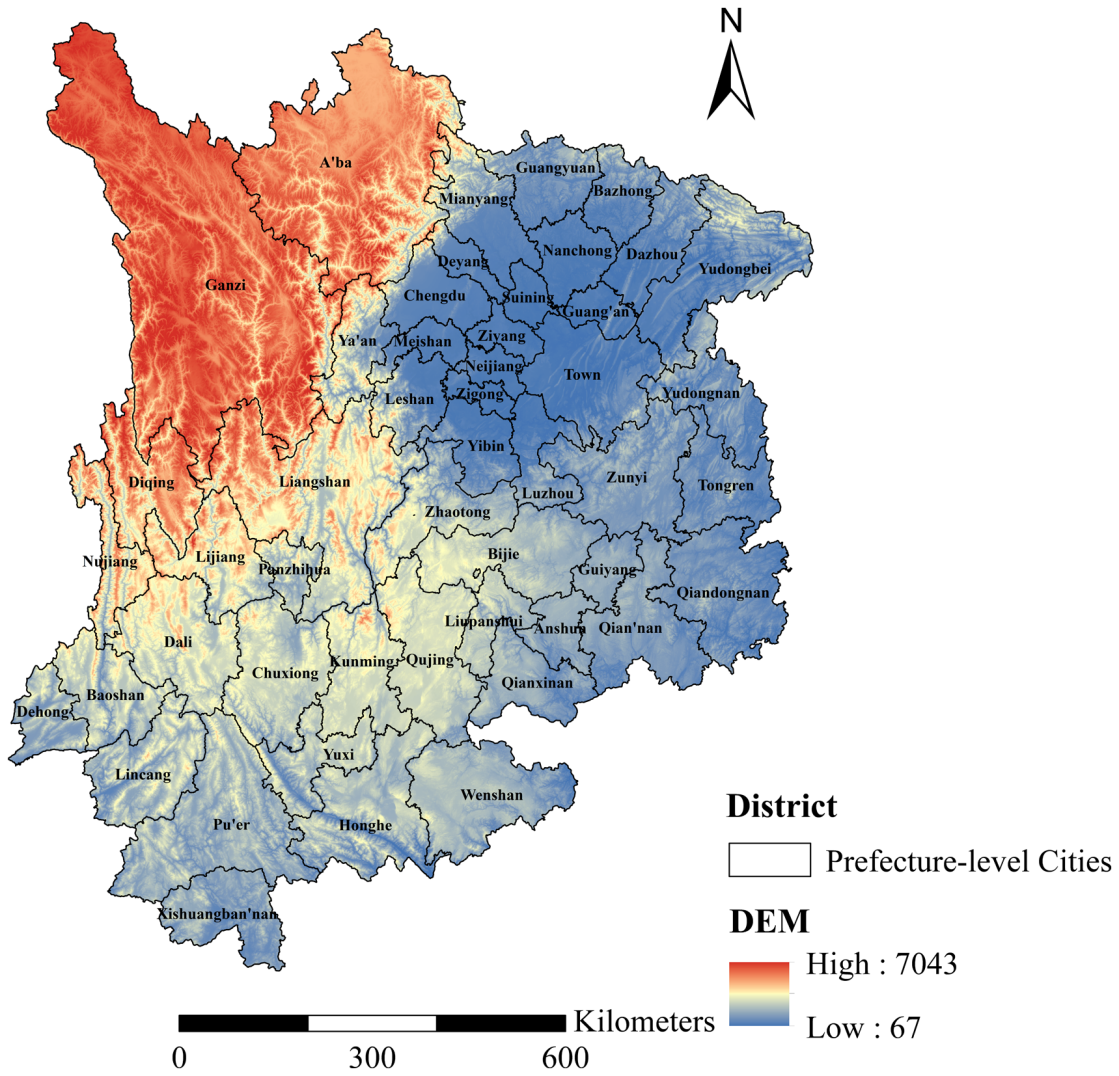
Study area.

Southwest China, comprising Yunnan Province, Guizhou Province, Sichuan Province and Chongqing Municipality, constitutes the study area of this research (See Figure 1). Located in the southwestern China, the region is characterized by complex terrain, diverse ethnic cultures and uneven socio-economic development.

Southwest China is dominated by mountainous and plateau landscapes, including the eastern extension of the Qinghai-Tibet Plateau and the Yunnan-Guizhou Plateau. Large variations in elevation, fragmented terrain and complex hydrological networks constraints infrastructure construction, transportation connectivity and spatial accessibility. These physical conditions have historically influenced settlement patterns and public service provision. The region also exhibits marked socio-economic disparities. Chengdu, Chongqing, Guiyang and Kunming function as major economic and population centers with relatively developed transportation and service systems, whereas many peripheral and mountainous areas experience more limited development and accessibility. This contrast provides an appropriate regional context for examining spatial inequality in public healthcare infrastructure.

Southwest China is also characterized by substantial ethnic and cultural diversity, including Miao, Dong, Yi and Tibetan communities as well as numerous traditional villages and cultural landscapes. Its abundant scenic attractions, parks, homestay accommodation and traditional villages provide spatial conditions associated with tourism-oriented residence and longer stays. Previous studies have reported growing tourism activity and the presence of seasonal or longer-stay visitors in parts of the region. However, the present study does not directly measure the size, duration or seasonal intensity of these population.

Accordingly, residential tourism areas are not treated as formally designated administrative zones. Instead, they are operationalized as tourism-related spatial environments identified through the concentration of A-level scenic spots, parks and squares, homestay accommodation and traditional villages. These indicators represent environments that may support residential tourism activities rather than direct observations of residential tourists or population mobility.

Taken together, the coexistence of topographic constraints, regional development disparities, concentrated urban centers and tourism-oriented spatial environments makes Southwest China a suitable setting for examining the spatial distribution of public healthcare infrastructure and its associations with environmental, socio-economic, residential and tourism-related factors.

## 4 Methodology

This study employed a cross-sectional spatial analytical framework to examine the distribution and associated factors of public healthcare infrastructure in Southwest China. Public healthcare infrastructure was represented by POI records of publicly operated general hospitals, primary healthcare institutions, community health service centers and township health centers, while private and other non-public medical institutions were excluded.

The methodological design was guided by the health geography perspective of spatial concentration, accessibility, regional inequality and place-based interaction. Average nearest neighbor analysis, Global Moran’s I and kernel density estimation were used to identify spatial distribution patterns, while the geographic detector model was applied to assess the explanatory power and interaction effects of environmental, socio-economic, residential and tourism-related variables. The analysis was designed to identify spatial associations rather than establish causal relationships.

### 4.1 Spatial analysis

Three complementary spatial methods were used to address the first research question and evaluate the spatial distribution pattern of public healthcare infrastructure. Average nearest neighbor analysis was applied to determine whether facility locations exhibited a clustered, dispersed or random point pattern (62). The nearest neighbor ratio compares the observed mean distance between each facility and its nearest neighbor with the expected distance under complete spatial randomness (63). A ratio below 1 indicates clustering, whereas a ratio above 1 indicates dispersion (64).

Global Moran’s I was used to assess whether healthcare infrastructure density exhibited significant overall spatial autocorrelation across the analytical units (65). Positive values indicate that areas with similar density levels tend to be spatially adjacent, negative values indicate spatial dissimilarity and values close to zero suggest a random spatial arrangement (66).

Kernel density estimation was used to transform facility points into a continuous density surface and visualize regional variations in concentration intensity (67). This method supports the identification of high-density cores, surrounding sub-high-density zones and extensive low-density areas. The search radius was generated using the default bandwidth calculation in QGIS 3.10 and the same parameter setting was applied across the entire study area to ensure regional comparability. The resulting classes represent relative density levels within a unified regional analysis.

### 4.2 Geographic detector

Residential tourism was operationalized through four tourism-related spatial proxy variables because direct data on residential tourist population size, length of stay and seasonal mobility were unavailable at the regional scale. These variables were selected to represent complementary destination conditions associated with longer stays and tourism-oriented residence. A-level scenic spot density captures the concentration of attractions that may support repeated or extended visits; park and square density reflect public leisure spaces used in everyday destination life; homestay accommodation density provides a more direct indication of tourism-oriented temporary lodging and residence; and traditional village density represents culturally distinctive rural environments in which residential tourism experiences may occur. The four indicators are therefore interpreted jointly as spatial characteristics of residential tourism environments rather than as direct measurements of residential tourists or equally strong proxies for long-stay residence.

The geographic detector model was used to address the second and third research questions. The factor detector assessed the explanatory power of individual variables, while the interaction detector examined whether pairs of variables enhanced or weakened their explanatory power when considered jointly (68). This design was used to evaluate whether transportation connectivity, residential density and tourism-related indicators exhibited stronger associations than natural environmental variables and whether interactions between accessibility and tourism-related factors increased explanatory power.

The study area was divided into regular 10 km x 10 km grid cells and each grid cell was treated as the basic spatial analytical unit (16). The grid system was adopted as a consistent regional analytical framework and was not intended to represent actual healthcare catchment areas or travel-time zones. All dependent and explanatory variables were processed within this unified grid system. The dependent variable was the grid-level spatial density of public healthcare infrastructure. Public healthcare infrastructure density was calculated for each 10 km x 10 km grid cell by counting the number of publicly operated general hospitals, public primary healthcare institutions, public community health service centers and public township health centers located within the cell and dividing the total count by the area of the grid cell. Because all regular grid cells had the same nominal area, the resulting density values primarily represented the number of public healthcare facilities per unit area. The four facility categories were combined into a single dependent variable and were treated as equally weighted point records because consistent regional data on hospital grade, bed capacity, healthcare personnel and service volume were unavailable.

The explanatory variables were grouped into three dimensions. Natural environment variables included slope and altitude. Socio-economic and residential variables included population density, GDP, nighttime illumination index, road network density, residential density and shopping centers and commercial supermarkets density. Tourism-related variables included A-level scenic spot density, park and square density, homestay accommodation density and traditional village density.

Point-based POI variables were converted into density values for each grid cell, road network density was calculated as total road length per unit area and raster-based variables were summarized within each grid cell using zonal statistics. The dependent variable was retained as a continuous numerical variable. All 12 variables were originally continuous and were discretized into five strata using Natural Breaks classification method in QGIS 3.10. This method minimizes within-class variance and maximizing between-class variance, allowing the classification to reflect the distributional structure of each variable (69).

The Geographic Detector evaluates whether the spatial stratification of an explanatory variable corresponds to the spatial variation of the dependent variable. Its q-statistic ranges from 0 to 1, with higher values indicating greater explanatory power for spatial differentiation. Interaction detection compares the joint q-value of two variables with their individual q-values to determine whether their combined explanatory power is enhanced, weakened or independent (70).

All spatial datasets were projected into a unified coordinate system and aligned with the same 10 km x10 km grid framework. POI records were cleaned through duplicate removal, coordinate correction, outlier exclusion and spatial matching. Raster datasets were clipped to the study area and resampled where necessary to ensure spatial consistency. The resulting grid-level dataset was used for both factor detection and interaction detection.

## 5 Data

### 5.1 Base map

The base map of administrative divisions for Southwest China was obtained from the China National Geographic Information Public Service Platform.^1^ Vector boundary data for Yunnan, Guizhou, Sichuan and Chongqing were downloaded from this platform and subsequently imported into QGIS 3.10 for further processing. To ensure spatial consistency and analytical accuracy, the geographic coordinate system of the datasets was first defined, followed by a transformation to an appropriate projected coordinate system suitable for spatial analysis. These preprocessing steps established a standardized spatial framework, providing a reliable basis for subsequent mapping and analytical procedures.

### 5.2 POI data

The dependent variable consisted exclusively of public healthcare institution including publicly operated general hospitals, public primary healthcare institutions, public community health service centers and public township health centers. Private hospitals, private clinics and other non-public medical institutions were excluded during data screening. This exclusion reflects the specific focus on publicly operated healthcare infrastructure rather than the complete healthcare provision market. The public or private status of each institution was identified based on POI category information, institutional names and available ownership-related descriptions. Only records that could be identified as public healthcare institutions were retained. The independent variable data include residential areas, commercial supermarkets, shopping centers, A-level scenic spots, parks, squares, homestays and traditional villages. All of these POI data are sourced from Amap.^2^ A program was written in Python 3.11 to obtain relevant POI data from Amap based on administrative divisions. The attributes of the obtained data include names, addresses and latitude-longitude coordinates. Subsequently a series of preprocessing steps are carried out on these data, including eliminating redundancy, correcting biases, excluding outliers and performing spatial matching. Finally, these data are imported into QGIS 3.10 to define the geographic coordinate system and projection coordinate system for visualization.

### 5.3 Road network data

The road network data used in this study were sourced from OpenStreetMap.^3^ The road network dataset covering China was downloaded from the platform and imported into QGIS 3.10 for preprocessing. Subsequently, the data were clipped to the spatial extent of the study area based on the administrative boundary base map of Southwest China. To ensure compatibility with other spatial datasets, projection transformation was performed to align the road network data with the unified coordinate system established for the study. These processing steps ensured the accuracy and consistency of the road network data for subsequent spatial analysis.

### 5.4 Remote-sensing data

This study utilizes remote-sensing data encompassing topographic elevation data, population data and nighttime illumination data, with specific data sources detailed as follows:

The topographic elevation data were obtained from the DEM dataset provided by the Institute of Geographic Sciences and Natural Resources Research, Chinese Academy of Sciences.^4^ The downloaded DEM raster data were imported into QGIS 3.10, where they were first clipped to match the spatial extent of the study area based on the administrative boundary. Subsequently, projection transformation was performed to ensure consistency with the unified coordinate system used in this study. Based on the processed DEM data, terrain variables including elevation and slope were further derived using spatial analysis tools in QGIS 3.10.

The population data utilized in this study were obtained from the LandScan global population dataset developed by Oak Ridge National Laboratory, U. S. Department of Energy.^5^ The global raster population data were imported into QGIS 3.10 and subsequently clipped to the spatial extent of Southwest China based on the administrative boundary of the study area. To maintain consistency with other spatial datasets, projection transformation was performed to align the data with the unified coordinate system adopted in this study.

The nighttime illumination data were obtained from the Visible Infrared Imaging Radiometer Suite (VIIRS) nighttime light dataset, with a spatial resolution of 500 m (71). The raster data were imported into QGIS 3.10 for preprocessing, where they were first clipped to the spatial extent of Southwest China based on the study area boundary. Subsequently, coordinate system definition and projection transformation were conducted to ensure consistency with the unified spatial reference system adopted in this study.

### 5.5 Panel data

The panel data employed in this study consist of Gross Domestic Product (GDP) statistics obtained from the National Bureau of Statistics of China.^6^ These data were first organized and standardized at the corresponding administrative unit level and them imported into QGIS 3.10 for spatial processing. Through spatial linkage with administrative boundary data, the attribute information was assigned to geographic units and subsequently converted into raster format using spatial interpolation or rasterization techniques.

## 6 Results

### 6.1 The result of spatial analysis

#### 6.1.1 The analysis of average nearest neighbor

This study employs the average nearest neighbor method to examine the spatial distribution characteristics of public healthcare infrastructure in Southwest China (see Table 1). The results show that the nearest neighbor ratio is 0.323, which is significantly less than 1, indicating a pronounced clustered distribution pattern. The *p*-value is 0.000 suggesting that the result is statistically significant at the 99% confidence level. In addition, the observed mean distance between components of public healthcare infrastructure is approximately 1,352 meters. This result indicates that public healthcare infrastructure is not randomly distributed but tends to concentrate in specific areas, reflecting an uneven spatial configuration of healthcare service provision across the region.

**Table 1:** Nearest neighbor analysis.

Ratio	Z-score	Mean Distance (m)	*p*-value	Type
0.323	−199.863	1352.644	0.000	Clustered

#### 6.1.2 The analysis of spatial autocorrelation

Building upon the preceding average nearest neighbor analysis, this study further applies spatial autocorrelation analysis to examine the spatial distribution characteristics of public healthcare infrastructure in Southwest China (see Table 2). The results show that the Global Moran’s I is approximately 0.582, which is significantly greater than 0 indicating a strong positive spatial autocorrelation. The *p*-value is 0.000, confirming statistical significance at the 99% confidence level. This finding suggests that areas with high concentrations of healthcare infrastructure tend to be spatially adjacent, while areas with low concentrations are also clustered together, further confirming the existence of spatial aggregation and regional disparity in healthcare infrastructure distribution.

**Table 2:** Global Moran’s I analysis.

Index	Z-score	*p*-value	Type
0.582	42.502	0.000	Clustered

#### 6.1.3 Kernel density analysis

**Figure 2:**
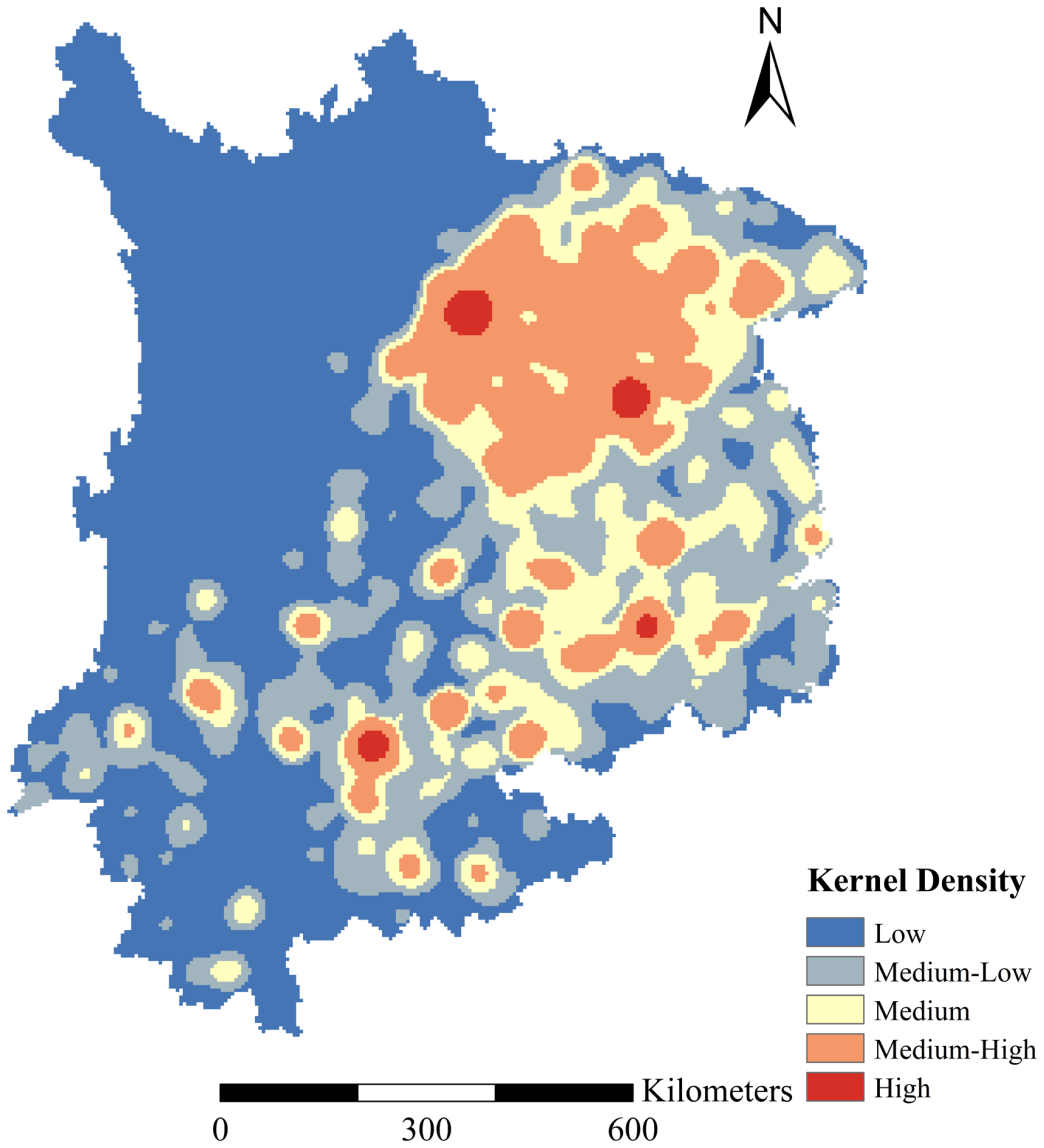
Kernel density analysis.

Based on the previous spatial analysis, it can be known that the spatial distribution characteristic of public healthcare infrastructure in Southwest China is agglomeration. In this section, kernel density analysis is employed to further explore the specific spatial distribution characteristics of public healthcare infrastructure (see Figure 2). According to the analytical results, it is evident that public healthcare infrastructure is predominantly clustered in the eastern part of the region, while the western part constitutes a relatively low-density area. Specifically, there are four high-density areas which correspond to the provincial capitals, namely Chengdu, Chongqing, Guiyang and Kunming. Among them, Chengdu and Chongqing serve as the cores giving rise to sub-high-density areas that spread outwards. In contrast, Guiyang and Kunming act as the cores forming concentric-circle shaped sub-high-density areas in their surrounding regions. Meanwhile, it is evident that there are several clustered distribution areas centered around sub-high-density regions. The prefectures and cities centered around these sub-high-density areas are Guangyuan City and Panzhihua City in Sichuan Province, Wanzhou District in Chongqing Municipality, Tongren City, Zunyi City, Bijie City, Liupanshui City, Panzhou City, Qiandongnan Miao and Dong Autonomous Prefecture, Qiannan Buyi and Miao Autonomous Prefecture and Qianxinan Buyi and Miao Autonomous Prefecture in Guizhou Province, as well as Qujing City, Zhaotong City, Baoshan City, Chuxiong Yi Autonomous Prefecture, Dali Bai Autonomous Prefecture, Honghe Hani and Yi Autonomous Prefecture and Wenshan Zhuang and Miao Autonomous Prefecture in Yunnan Province. While, extensive low-density areas are observed in Aba Tibetan and Qiang Autonomous Prefecture, Ganzi Tibetan Autonomous Prefecture and Liangshan Yi Autonomous Prefecture in Sichuan Province, as well as in Diqing Tibetan Autonomous Prefecture, Zhenyuan Yi, Hani and Lahu Autonomous County, Jinggu Dai and Yi Autonomous County, Mojiang Hani Autonomous County and Mengla County in Yunnan Province. This pattern reflects a pronounced spatial imbalance in facility concentration, with public healthcare institutions clustered in major urban cores and lower facility density observed in many peripheral regions. Because the analysis is based on POI locations, these patterns should not be interpreted as direct measures of healthcare service capacity or actual population coverage.

### 6.2 The result of geographic detector

#### 6.2.1 The result of selected detector factors

Building upon previous research and embodied observations (72–78), this study selected three dimensions encompassing 12 influencing factors as independent variables to conduct a geographic detection into the driving intensity of spatial distribution among public healthcare infrastructure in Southwest China. The selected elements are shown in Table 3.

**Table 3:** Influencing factors.

Dimension	Factor
Nature environment	Terrace Slope (X1)
	Terrace Altitude (X2)
Society economic	Population Density (X3)
	Gross Domestic Product (X4)
	Nighttime Illumination Index (X5)
	Road Network Density (X6)
	Residential Density (X7)
	Density of Shopping Center and Commercial Supermarket (X8)
Tourism resource	A-level Scenic Spots Density (X9)
	Density of Park and Square (X10)
	Homestay Accommodation Density (X11)
	Traditional Village Density (X12)

#### 6.2.2 The results of single-factor detector

The results of the single-factor detection are presented in Table 4. It is evident that the *p*-value of X1 to X11 are all 0.000, indicating that these factors pass the 99% significance test. Meanwhile, the *p*-value of X12 is 0.022 which also passes the 95% significance test. Therefore, all selected factors have statistically significant effects on the spatial distribution of public healthcare infrastructure in Southwest China. In terms of explanatory power, road network density (X6) has the highest q-value, indicating that transportation connectivity is the most important factor influencing the spatial distribution of public healthcare infrastructure. This result suggests that healthcare infrastructure is more likely to be located in areas with better road connectivity, because such areas provide more favorable locational conditions for healthcare facilities and may be associated with easier geographic access. In Southwest China, where mountainous terrain and fragmented landscapes are prominent, road networks directly affect the accessibility of healthcare services. Therefore, areas with weak road connectivity tend to exhibit lower concentrations of public healthcare facilities. A-level scenic spot density (X9) ranks second, indicating that tourism-related spatial environments are strongly associated with the distribution of public healthcare infrastructure. Because the study does not include direct tourist-flow or mobile-population data, this result should not be interpreted as evidence that population mobility itself determines healthcare infrastructure allocation. Rather, A-level scenic spot density represents the spatial concentration of tourism resources and may indicate areas where tourism-oriented service environments overlap with residential and public service functions. The observed association therefore reflects spatial co-location rather than a directly measured mobility effect. Residential density (X7) also shows strong explanatory power, ranking third among all factors. This result indicates that the spatial distribution of healthcare infrastructure remains closely related to the concentration of daily living spaces and permanent population. Areas with higher residential density usually have more stable healthcare demand, which makes them more likely to attract or support the establishment of healthcare infrastructure. This also explains why healthcare infrastructure tends to concentrate in urbanized and densely inhabited areas rather than in sparsely populated mountainous regions. In addition, the density of parks and squares (X10), shopping centers and commercial supermarkets (X8), nighttime illumination index (X5) and population density (X3) also show relatively strong explanatory power. These factors are closely associated with urban development, public activity intensity and socio-economic vitality. Their influence suggests that public healthcare infrastructure is not only distributed according to population size, but also closely linked to broader urban service functions and regional development levels. In other words, areas with stronger economic activities and more developed urban service functions tend to exhibit higher concentrations of public healthcare facilities.

**Table 4:** Single-factor driving influence.

Influence Significance	X1	X2	X3	X4	X5	X6
*q*	0.096	0.139	0.200	0.089	0.273	0.610
*p*	0.000	0.000	0.000	0.000	0.000	0.000

Influence Significance	X7	X8	X9	X10	X11	X12
*q*	0.416	0.342	0.521	0.356	0.169	0.002
*p*	0.000	0.000	0.000	0.000	0.000	0.022

By contrast, altitude (X2), slope (X1), GDP (X4) and traditional village density (X12) show relatively weaker explanatory power. The relatively low q-values of altitude and slope do not mean that natural conditions are unimportant; rather their influence may be indirectly reflected through road construction, settlement distribution and economic development. In mountainous regions, topographic constraints often affect healthcare infrastructure distribution by limiting transport accessibility and increasing construction costs. Similarly, the low explanatory power of traditional village density suggests that cultural heritage resources alone do not directly determine healthcare infrastructure distribution. Their influence may depend on whether these villages are effectively connected with tourism development, transportation networks and residential demand. Traditional village density (X12) has the lowest explanatory power with a q-value of only 0.002. Although its *p*-value indicates statistical significance, its substantive contribution to the spatial differentiation of public healthcare infrastructure is negligible. Therefore, traditional village density should not be regarded as an important independent determinant of healthcare infrastructure distribution in Southwest China.

Overall, the single-factor results show that transportation connectivity, tourism-related spatial concentration and residential distribution are the principal factors associated with public healthcare infrastructure. By contrast, traditional village density contributes almost no independent explanatory power and is therefore not treated as a substantive determinant in the subsequent interpretation.

#### 6.2.3 The results of the interaction detector

**Figure 3:**
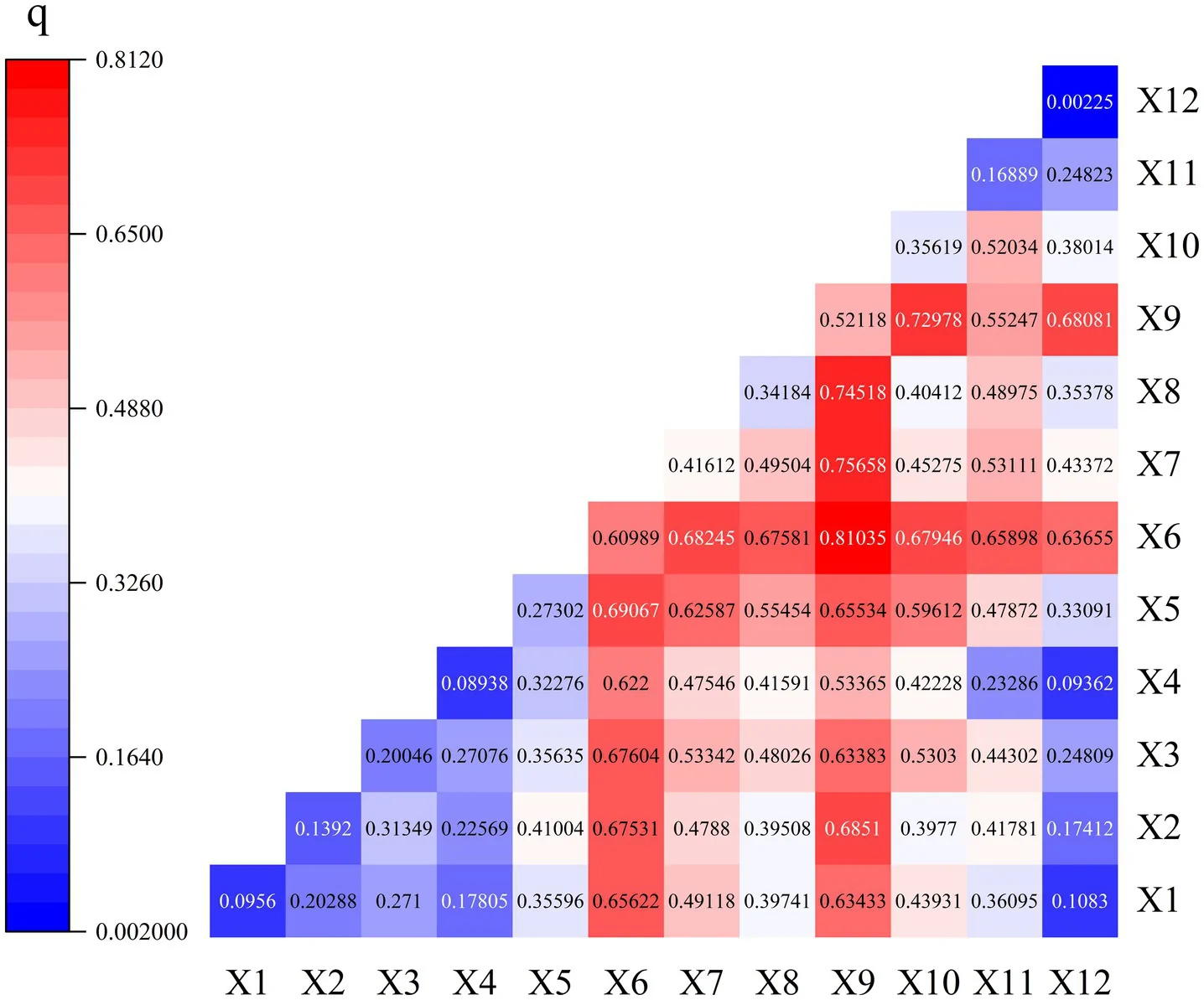
Interaction detector.

Building upon the investigation of single-factor influences, this study employs interaction detection analysis to further explore the driving intensity of mutual interactions among the determinants of the spatial distribution of public healthcare infrastructure in Southwest China (see Figure 3). The first numerical value in each row of the figure indicates the explanatory power of a single factor, while the remaining values represent the interaction effects between pairs of factors. The results show that the interaction effects among all pairs of influencing factors are stronger than the explanatory power of individual factors. This indicates that the spatial distribution of public healthcare infrastructure is not shaped by a single determinant, but by the combined effects of multiple factors. In other words, transportation connectivity, tourism development, population distribution, urban service functions and natural environmental conditions jointly influence the allocation of healthcare infrastructure.

Among all interaction pairs, the combination of road network density (X6) and A-level scenic spot density (X9) shows the strongest interaction effect. This finding indicates that transportation connectivity and tourism-related spatial environments jointly exhibit strong explanatory power for the distribution of public healthcare infrastructure. Areas with both dense road networks and concentrated tourism resources may provide more favorable conditions for facility location, service delivery and the spatial co-location of tourism, residential and public service functions. However, because no direct mobility indicators are included, this interaction should not be interpreted as evidence of mobility-driven healthcare demand. At the same time, good road connectivity improves the feasibility of facility construction and service delivery. Therefore, the interaction between road networks and scenic spots reflects a strong spatial association between transportation connectivity and tourism-oriented environments rather than a directly measured mobility-driven demand mechanism. In addition, the relatively strong interactions between A-level scenic spot density (X9) and residential density (X7), shopping center and commercial supermarket density (X8), and park and square density (X10) suggest that tourism-related spatial indicators have greater explanatory power when they overlap with residential and urban service environments. These results indicate that spatial co-location among tourism resources, daily living spaces and public service functions rather than directly measured mixed demand from mobile populations. In residential tourism areas, these interaction results indicate the spatial overlap of tourism resources, residential areas and urban service functions. Because the model does not include residential tourist population size or seasonal stay intensity, the findings should not be interpreted as direct evidence of mixed or seasonal healthcare demand. Therefore, these interaction results reveal the mixed-demand characteristics of healthcare services in tourism-oriented regions. The interaction between road network density (X6) and nighttime illumination index (X5) also shows a strong effect, suggesting that transportation connectivity and regional development intensity jointly influence healthcare infrastructure distribution. Nighttime illumination generally reflects the level of urbanization, economic activity and public service concentration. When areas with strong economic vitality are also supported by good transportation networks, they are more capable of attracting and sustaining healthcare facilities. This explains why healthcare infrastructure tends to cluster in urbanized and economically active areas rather than in remote mountainous regions with weak accessibility. It is also noteworthy that some natural and cultural factors show stronger influence when interacting with tourism or transportation variables. The interaction between traditional village density (X12) and A-level scenic spot density (X9) shows a higher explanatory value than traditional village density alone. However, because the single-factor q-value of X12 is only 0.002, this result should be interpreted cautiously. It indicates a conditional statistical enhancement when traditional village density is considered together with scenic spot density rather than evidence that traditional villages independently or substantially shape healthcare infrastructure distribution. This result is particularly relevant for Southwest China, where mountainous landscapes, ethnic villages and tourism development are closely connected.

In contrast, the interaction between GDP (X4) and traditional village density (X12) shows the weakest interaction effect. This further confirms that traditional village density contributes little explanatory power to the spatial distribution of public healthcare infrastructure, either independently or in combination with general economic scale.

Overall, the interaction detector results reveal nonlinear enhancement among accessibility, tourism-related spatial indicators, residential distribution and regional development conditions. These findings indicate that healthcare infrastructure distribution reflects the combined spatial associations of transportation networks, tourism-oriented environments, residential concentration and urban service functions. They should not be interpreted as direct evidence of population mobility or mobile healthcare demand.

## 7 Discussion

This study applies a health geography framework to examine public healthcare infrastructure as a spatially embedded public service system. From this perspective, the distribution of healthcare facilities reflects the combined influence of geographic accessibility, population and residential distribution, regional development conditions and place-specific spatial environments. The findings should be interpreted within a cross-sectional analytical framework. Specifically, the study identifies spatial clustering, core-periphery disparities and explanatory associations between healthcare infrastructure distribution and selected environmental, socio-economic and tourism-related spatial indicators. Rather than identifying causal or dynamic mobility mechanisms, the analysis demonstrates how healthcare infrastructure distribution is spatially associated with accessibility, regional inequality and tourism-oriented environments.

### 7.1 Spatial distribution characteristics

The results demonstrate that public healthcare infrastructure in Southwest China exhibits a significant clustered spatial pattern, as confirmed by both the average nearest neighbor index (R = 0.323) and Moran’s I (0.582). From the perspective of health geography, such clustering reflects spatial unevenness in the geographic distribution of healthcare facilities and may have implications for healthcare accessibility (79), which is closely linked to the distribution of population, economic resources and transportation networks.

Kernel density analysis further reveals a clear core-periphery structure with high-density clusters concentrated in major urban centers such as Chengdu, Chongqing, Guiyang and Kunming, while extensive low-density areas are distributed across remote mountainous regions. This pattern is consistent with the fundamental principle in health geography that healthcare resources tend to agglomerate in areas with higher demand intensity and stronger service capacity (80), while peripheral regions often exhibit lower concentrations of healthcare facilities due to geographic and economic constraints (81).

Importantly, this spatial configuration also indicates that healthcare accessibility is not only a function of facility quantity but is inherently shaped by spatial proximity, transport connectivity and terrain conditions (82), all of which are central concerns in health geography research. In mountainous regions such as Aba Tibetan and Qiang Autonomous Prefecture in Sichuan, physical barriers and fragmented landscapes may constrain geographic access to existing facilities, thereby reinforcing spatial inequality in healthcare provision.

### 7.2 Spatial associations and interaction patterns

The geographic detector results show that road network density (X6), A-level scenic spot density (X9) and residential density (X7) exhibit the strongest spatial associations with the distribution of public healthcare infrastructure. From a health geography perspective, these findings indicate spatial correspondence among transportation connectivity, residential concentration, tourism-oriented environments and healthcare facility distribution. However, the geographic detector identifies spatial stratified heterogeneity rather than causal direction (83) and the observed relationships may reflect reciprocal processes or the common influence of urbanization and regional development.

First, road network density exhibits the strongest explanatory power, underscoring the close association between transportation connectivity and healthcare facility location. In health geography, accessibility is considered a key determinant of healthcare equity, as transportation networks directly affect the ability of populations to reach medical services (84, 85). Although actual travel time or service utilization was not measured, the result suggests that transportation connectivity is an important locational correlate of facility concentration. The high q-value of road network density indicates that areas with dense road networks also tend to exhibit higher concentrations of public healthcare facilities. This spatial correspondence may reflect the locational advantages provided by transportation connectivity, but it may also arise because road networks, healthcare facilities and other public services develop jointly during urban expansion. The present analysis cannot determine whether road development precedes healthcare facility location or whether both are shaped by broader regional development processes.

Second, the influence of tourism-related factors, particularly A-level scenic spot density suggests that tourism-oriented spatial environments are associated with the distribution of public healthcare infrastructure. It should be noted that no direct tourism mobility variable was included in the model. The tourism-related variables used in this study represent spatial characteristics of tourism-oriented environments rather than actual tourist flows or seasonal population movements. These indicators are meaningful because residential tourism depends not only on accommodation, but also on destination environments that support repeated visits, everyday leisure and temporary residence (86). Homestay accommodation is more directly related to tourism-oriented lodging, whereas scenic spots, parks and traditional villages mainly represent attraction, leisure and place-based environmental conditions. Accordingly, the strong explanatory contribution of A-level scenic spot density should be interpreted as an association with tourism-oriented destination concentration rather than as direct evidence of long-stay tourist residence. The observed association may also reflect the co-location of scenic spots, commercial services, transport infrastructure and healthcare facilities in more urbanized or developed destinations (87). Therefore, it should not be interpreted as evidence that tourism activity or residential tourists directly generate healthcare facility development.

Third, the interaction detector results indicate that the effects of influencing factors are not independent but exhibit nonlinear enhancement relationships, particularly between transportation and tourism variables (e.g., road density with scenic spots). This finding is consistent with the health geography emphasis on interacting spatial conditions (11). Nevertheless, the enhanced q-values indicate stronger spatial correspondence when variables are considered jointly and do not establish a causal mechanism or temporal sequence. The interaction between road density and scenic spot density may reflect their shared concentration in developed urban and tourism-oriented areas rather than a direct process through which tourism demand generates healthcare facilities.

### 7.3 Health geography interpretation

The theoretical contribution of this study lies primarily in extending the application of health geography to residential tourism contexts rather than proposing a new health geography theory. Existing health geography research on healthcare infrastructure has largely focused on permanent resident populations, urban–rural disparities and conventional socio-economic determinants (88, 89). This study adds tourism-oriented spatial environments to this framework and examines their associations with healthcare facility distribution alongside transportation connectivity, residential density and regional development conditions. The residential tourism context in this study is therefore conceptualized as a combination of destination attractions, tourism-oriented accommodation and everyday leisure environments rather than as a population category directly observed in the data.

The concentration of healthcare facilities in major urban centers and the strong association with road network density are broadly consistent with previous studies of healthcare infrastructure in China (90). These findings are therefore not presented as entirely new spatial regularities. The contribution of the present study lies instead in examining these established relationships within tourism-oriented environments and in testing how transportation, residential and tourism-related spatial indicators jointly correspond to public healthcare facility distribution. In particular, the interaction analysis shows that tourism-related indicators have greater explanatory relevance when considered together with road connectivity and residential concentration, although these relationships remain spatial associations rather than evidence of seasonal or mobile healthcare demand.

This extension highlights that place-based healthcare analysis may need to consider not only conventional residential and economic spaces but also tourism-oriented environments that overlap with local public service systems (91). The findings do not demonstrate direct mobility effects, but they show that tourism-related spatial indicators have explanatory relevance when considered together with accessibility and residential distribution. In this sense, the study broadens the contextual scope of health geography analysis while retaining its core concern with spatial inequality, accessibility and place-based interaction. Among the tourism-related indicators, A-level scenic spot density provides the strongest explanatory contribution, whereas traditional village density has negligible independent explanatory power. Therefore, the tourism-related findings are driven primarily by scenic spot concentration and other tourism-supporting spatial environments rather than by traditional village distribution itself.

This study attempts to extend health geography analysis to residential tourism areas, where healthcare accessibility needs to be interpreted in relation to both permanent residential spaces and tourism-oriented spatial environments. In such regions, tourism resources, homestay accommodation areas and traditional village may indicate potential temporary users of local services (92), although actual tourists flows and seasonal population movements are not directly measured in this study.

From a health geography perspective, this finding highlights the need to consider tourism-related spatial contexts when interpreting healthcare accessibility. The present study does not measure temporal fluctuations in healthcare demand. Instead, it shows that tourism-related spatial indicators, when combined with residential density and transportation connectivity are associated with the spatial distribution of healthcare infrastructure. Although areas with concentrated tourism resources may face potential additional service pressure, the present study does not directly measure periodic changes in healthcare demand. Future research should incorporate tourism flow data, seasonal population data or healthcare service-utilization records to examine whether such temporal demand changes occur.

The very low q-value of traditional village density (X12) indicates that it has almost no independent explanatory power for the spatial distribution of public healthcare infrastructure. Although its interaction with A-level scenic spot density shows statistical enhancement, this result does not establish a substantive indirect mechanism. Therefore, traditional village density is treated as a marginal contextual variable rather than a core explanatory factor in the health geography interpretation.

### 7.4 Policy and practical implications

The findings of this study provide a preliminary spatial screening reference for healthcare planning in tourism-oriented environments. Because the analysis does not measure healthcare utilization, facility capacity, seasonal population change or actual service overload, the results should not be used independently to determine the location or scale of new healthcare investments. Policy priorities should vary according to transportation connectivity, existing facility concentration, residential distribution and tourism-related spatial conditions.

For remote mountainous and low-density areas, priority should be given to improving basic healthcare accessibility rather than simply increasing the number of fixed facilities. Provincial and prefecture level health authorities could identify counties with low facility concentration and weak road connectively for further capacity and demand assessment. In areas where permanent facility construction is constrained by terrain and dispersed settlements, practical measures may include upgrading township health centers, establishing referral links with county-level general hospitals, deploying mobile medical teams and improving emergency transport routes. Road improvement projects should also prioritize connections between remote settlements and existing healthcare service centers.

For areas where tourism resources, residential spaces and transport networks are highly concentrated, healthcare planning may use tourism-related spatial indicators as supplementary screening information. Local health and tourism authorities could jointly identify locations where A-level scenic spots, homestay accommodation, parks, traditional villages and residential areas spatially overlap with relatively low concentrations of public healthcare facilities. These locations should be treated as candidate areas for further investigation rather than as direct priorities for new healthcare investment. Before any intervention is considered, local authorities should verify actual need using seasonal visitor numbers, healthcare utilization records, emergency case data, facility capacity, road travel time and permanent resident demand. Only where additional demand or service pressure is empirically confirmed should measures such as temporary medical support, strengthened emergency referral arrangements, expanded primary healthcare capacity or improved medical information service be considered. Because the present study does not directly measure seasonal demand or service overload, no resource-allocation decision should be made solely on the basis of spatial overlap or proxy indicators.

For major urban cores such as Chengdu, Chongqing, Guiyang and Kunming, policy attention should focus less on the total number of facilities and more on service coordination, hierarchical referral and the spatial balance between central and peripheral districts. In contrast, surrounding sub-high-density and low-density areas should receive priority in the expansion of primary healthcare coverage. Regional planners could use the kernel density results to distinguish highly concentrated urban cores from peripheral areas with lower facility density and avoid the repeated concentration of new facilities in already well-served central areas.

To improve implementation, a cross-sector spatial planning mechanism should be established among health, transportation, tourism and local planning departments. A shared GIS-based database could be used to regularly update healthcare facilities, road accessibility, residential distribution and tourism-related spatial indicators. These data could support the identification of priority intervention areas, the evaluation of proposed facility location and supporting the preliminary identification of areas requiring additional capacity, utilization and travel-time assessment. Such a mechanism would translate the interaction effects identified in this study into a practical planning tool.

Overall, the results should be used as an initial spatial screening framework rather than a direct basis for healthcare resource allocation. Areas characterized by low facility concentration, weak road connectivity or overlap between tourism-oriented environments and residential spaces may warrant further investigation. Final planning decisions should be based on verified evidence from facility capacity, road travel time, healthcare utilization, seasonal visitor volumes, permanent resident demand and community-level survey. This evidence-based verification is necessary to avoid directing public investment toward locations where spatial overlap does not correspond to actual healthcare need.

### 7.5 Research limitations and future research directions

While this study provides an initial examination of the spatial distribution and determinants of public healthcare infrastructure in residential tourism areas, several limitations should be acknowledged.

First, residential tourism was represented through tourism-related spatial environments rather than direct observations of long-stay or semi-permanent tourist populations. The selected indicators capture different destination conditions associated with tourism-oriented residence: scenic spots represent destination attractions, parks and squares represent everyday leisure environments, homestays represent tourism-oriented accommodation, and traditional villages represent culturally distinctive rural settings. However, these indicators do not have equally direct relationship with residential tourism. In particular, scenic spot density and traditional village density may also reflect conventional sightseeing tourism resource distribution. The model does not include overnight visitor numbers, average length of stay, repeated-visit frequency, accommodation occupancy or seasonal residence proportions. Therefore, the findings should not be interpreted as direct evidence of residential tourist population size, long-stay intensity or tourism-generated healthcare demand. Future research should incorporate overnight-stay statistics, accommodation nights, seasonal population data, mobile signaling data or visitor surveys to distinguish short-term sightseeing tourism from longer-stay residential tourism more directly.

Second, the POI-based healthcare dataset primarily captures the spatial presence and concentration of facilities rather than their functional capacity. Publicly operated general hospitals, primary healthcare institutions, community health service centers and township health centers were treated as point records in the spatial analysis. Consequently, facilities with substantial differences in hospital grade, bed capacity, number of physicians, equipment, service volume and functional specialization contributed equally to the density surface. This equal-weight treatment may cause areas containing numerous small facilities to appear highly concentrated, while areas containing fewer but higher-capacity institutions may appear less prominent. The results therefore represent facility distribution and concentration rather than actual healthcare supply, service quality, population coverage or effective accessibility. Future research should integrate hospital grade, bed numbers, healthcare personnel, equipment, outpatient volume and service catchment data and employ capacity-weighted density analysis, two-step floating catchment area methods or network-based accessibility models.

Third, the study focuses exclusively on publicly operated healthcare institutions. Private hospitals, private clinics, rehabilitation centers, wellness institutions, residential care facilities and tourism-oriented medical providers were excluded because the study was designed to examine public healthcare infrastructure rather than the complete regional healthcare market. Nevertheless, these institutions may constitute important components of healthcare provision in tourism-oriented destinations, particularly where medical examination, rehabilitation, traditional Chinese medicine, wellness and long-term residence are integrated. Their exclusion limits the ability of the present analysis to portray the full landscape of healthcare provision available to tourists, retirees and other temporary residents. Future studies should compare public and private providers and distinguish among general medical care, rehabilitation, wellness, residential care and tourism-oriented healthcare functions.

Fourth, the regular 10 km x10 km grid provides a consistent regional analytical unit across Southwest China but may smooth fine-scale spatial variation and geographic barriers within mountainous areas. A grid cell does not represent an actual healthcare catchment area and conditions within the same cell may vary substantially because of elevation differences, rivers, steep slopes and discontinuous road connections. In addition, road network density was used as an indicator of transportation connectivity but does not directly measure actual travel time, route impedance or cost distance. A straight-line distance of 10 km may correspond to markedly different travel conditions in mountainous and urban areas. Accordingly, the findings concerning transportation connectivity should be understood as grid-level spatial associations rather than direct measurements of healthcare travel accessibility. Future research should compare multiple grid resolutions and apply road-network travel-time analysis, terrain-adjusted cost-distance modeling and service catchment approaches.

Fifth, although elevation and slope were incorporated into the geographic detector model, they were represented as grid-level numerical variables and may not fully capture the discontinuous barrier effects of mountainous terrain on movement and healthcare access. Climatic conditions such as temperature, humidity, precipitation and seasonal climatic comfort were also not included. These factors may be particularly relevant to longer-stay tourism in Southwest China, where vertical climatic variation and seasonal environmental comfort may influence destination choice and residence duration. The current variable system should therefore be understood as an analysis of factors associated with healthcare facility distribution rather than a comprehensive model of residential tourism attractiveness or seasonal residence behavior. Future research should incorporate terrain-adjusted accessibility indicators, climatic comfort indices and seasonal tourism data.

In addition, the geographic detector model identifies spatial stratified heterogeneity and interaction enhancement but does not establish causal direction or temporal sequence. The observed associations may reflect reciprocal relationships or the common influence of urbanization, regional development and public investment. For example, road networks and commercial services may facilitate healthcare facility location, but healthcare and urban expansion may also stimulate surrounding infrastructure development. Future longitudinal research should employ panel data, quasi-experimental designs or spatial regression approaches to examine temporal ordering and potential causal mechanisms.

Finally, the study adopts a cross-sectional design and focuses on Southwest China. It cannot identify temporal changes in healthcare facility distribution, tourism activity or service demand and the findings may not be directly generalizable to regions with different terrain, development conditions or healthcare systems. Comparative and longitudinal studies using panel data, healthcare utilization records and multi-regional samples would help assess the temporal stability and broader applicability of the observed spatial relationship.

## 8 Conclusion

Based on the analysis of spatial distribution patterns and influencing factors of public healthcare infrastructure in residential tourism areas of Southwest China, several key findings can be summarized as follows:

(1) Public healthcare infrastructure exhibits a significant clustered spatial distribution pattern, with a clear core–periphery structure. High-density clusters are mainly concentrated in major urban centers, while low-density areas are widely distributed across remote mountainous regions, reflecting pronounced spatial inequality in facility distribution and potential geographic access.
(2) Road network density, A-level scenic spot density and residential density emerge as the primary factors influencing the spatial distribution of healthcare infrastructure. Among them, transportation connectivity plays a particularly critical role in shaping the spatial configuration of healthcare services.
(3) The interaction effects between influencing factors demonstrate nonlinear enhancement relationships, especially between transportation and tourism-related variables. This suggests that the spatial distribution of healthcare infrastructure is the result of multi-factor coupling rather than single-factor influence.
(4) In the context of residential tourism, tourism-related spatial indicators are associated with the distribution of public healthcare infrastructure. However, these indicators represent tourism-oriented spatial environments rather than directly observed population mobility or temporal healthcare demand.

Overall, the spatial distribution of public healthcare infrastructure in Southwest China reflects the combined associations of natural conditions, socio-economic development, transportation connectivity, residential distribution and tourism-related spatial environments. These findings may provide a reference for supporting more spatially balanced facility planning and identifying areas requiring further service-capacity assessment. in regions undergoing rapid tourism development. From a theoretical perspective, the study extends the application of health geography by incorporating tourism-related spatial environments into an analytical framework centered on facility distribution, transportation connectivity, residential concentration and place-based interaction. The contribution should be understood as a contextual and integrative extension rather than the identification of entirely new spatial regularities or direct evidence of seasonal healthcare demand.

## Data Availability

The original contributions presented in the study are included in the article/supplementary material, further inquiries can be directed to the corresponding author/s.

## Footnotes

1 https://www.tianditu.gov.cn, accessed on 20 January 2026

2 http://www.amap.com, accessed on 20 January 2026

3 https://www.openstreetmap.org, accessed on 21 January 2026

4 https://www.resdc.cn, accessed on 21 January 2026

5 https://landscan.ornl.gov, accessed on 21 January 2026

6 https://data.stats.gov.cn, accessed on 22 January 2026
